# Risk factors analyses for lateral lymph node metastases in papillary thyroid carcinomas: a retrospective study of 356 patients

**DOI:** 10.1590/2359-3997000000218

**Published:** 2016-09-26

**Authors:** Xilin Nie, Zhuo Tan, MingHua Ge, LieHaoJiang, JiaFeng Wang, ChuanMing Zheng

**Affiliations:** 1 Department of Head and Neck Surgery Zhejiang Cancer Hospital China Department of Head and Neck Surgery, Zhejiang Cancer Hospital, 38 Guangji Road, Hangzhou, 310022, China

**Keywords:** Papillary thyroid carcinoma, lateral lymph node metastasis, central lymph node metastasis, extrathyroidal extension, risk factors

## Abstract

**Objective:**

The aim of this study was to investigate the incidence and risk factors for lateral lymph node metastasis (LLNM) in patients with papillary thyroid carcinoma (PTC).

**Subjects and methods:**

356 patients diagnosed with PTC who underwent total thyroidectomy and central lymph node dissection and lateral lymph node dissection between January 2005 and December 2011 were enrolled. The relation between LLNM and clinicopathological features such as gender, age, tumor size, tumor spread, psammoma bodies, tumor multifocality, extrathyroidal extension (ETE), unilateral or bilateral disease, tumor primary location and central lymph node metastases (CLNM) was analyzed.

**Results:**

The rate of LLNM was 75.0%. In the univariate analysis, it was significantly associated with age, tumor size, tumor spread, extrathyroidal extension, primary tumor location and central lymph node metastasis (p < 0.05). In contrast, in the multivariate analysis, it was significantly associated with primary tumor location, central lymph node metastasis (p < 0.05) and tumor size > 1.5 cm with p = 0.05 but was unrelated to the other factors.

**Conclusion:**

Patients with PTC, with the primary tumor located in the upper part of the lobe and positive central compartment lymph node metastasis with a tumor size > 1.5 cm diameter are more likely to have LLNM. Therefore, more meticulous evaluations including the lateral lymph nodes should be performed before surgery.

## INTRODUCTION

The incidence of thyroid carcinoma is increasing by 4% per year. Papillary thyroid carcinoma (PTC) accounts for approximately 80% of all thyroid cancers and 1% of all human malignancies (1). It is the most likely of all thyroid malignancies to metastasize to the cervical lymph nodes, with 18%-90% of patients developing metastasis to the cervical region, and it is associated with a higher rate of locoregional recurrence and distance metastasis (2-4). Despite the high metastasis rate, PTC still has an excellent prognosis as an indolent disease (5). Growing evidence suggests that lymph node metastasis adversely affects survival, particularly in older patients with large tumors and extrathyroidal extensions (6). In addition, the risk of regional recurrence is higher in patients with cervical lymph node metastasis, especially in those with more than 10 involved nodes and extracapsular extensions (7).

Generally, the cervical lymph node metastasis of PTC was found to occur first in the central compartment (level VI) and then spread to the lateral compartment of the neck (8). New evidence from a large-scale nested case-controlled study suggests that patients with lymph node metastasis experience a higher mortality, and incomplete surgical excision is an important reason for the increased mortality in patients with stage I PTC (6). Therefore, in spite of the controversy over treatment, current guidelines propose prophylactic ipsilateral central compartment dissection for patients with PTC (9).

Prophylactic lateral neck dissection is not generally recommended (9). There is a general consensus that if there is any certain evidence for lateral lymph node metastasis in the preoperative evaluation for PTC based on physical examination findings, imaging, cytology, or intraoperative assessment, then a therapeutic lateral neck dissection should be performed (9). However, the extent of therapeutic lateral neck dissection remains controversial. Overall, there is no evidence to prove which approach is most appropriate for the management of lateral compartment lymph node metastasis, although most specialists believe that “berry-picking” should be avoided for the initial therapeutic lateral neck dissection, and a compartment-oriented en-bloc lateral neck dissection should be advocated (9,10).

The aim of this study was to investigate the incidence and risk factors of lateral lymph node metastasis in PTC with preoperative suspicious cN+. We retrospectively reviewed clinical data from patients in our hospital treated with a systematic, therapeutic lateral neck dissection.

## SUBJECTS AND METHODS

We retrospectively reviewed the clinical records of 356 patients with PTC who underwent total thyroidectomy and central lymph node dissection and lateral lymph node dissection if the preoperative evaluation suggested lateral node metastasis. The enrolled patients were treated between January 2005 and December 2011 and received the first treatment in the Department of Head and Neck Surgery, Zhejiang Cancer Hospital. All the patients were diagnosed with PTC with LLNM by general pathological examination in the Department of Pathology, Zhejiang Cancer Hospital. Patients with other types of thyroid malignancy or with tumors in the isthmus were excluded. Patients with a history of neck surgery for other diseases or radiation exposure were excluded.

All procedures and studies involving human participants were in accordance with the ethical standards of the institutional and/or national research committee and the 1964 Helsinki Declaration and its later amendments or comparable ethical standards. This study was approved by the Institutional Review Board of Zhejiang Cancer Hospital (No. IRB-2015-245), and for this type of study, formal consent is not required.

All the patients underwent physical examination (PE), thyroid gland and neck lymph node ultrasonic examination (US), and neck and thorax contracted computer tomography (CT). Fine needle aspiration cytology was not systematically performed in our hospital at time of the study. The criteria for metastasis requiring US were as follows: round shape (long/short ratio < 2), micro-calcification, cystic change, hyperechogenicity and heterogeneous inner structure (11). The criteria for CT were as follows: enhancement, heterogeneous, cystic or necrotic change and round shape. The size criteria for both US and CT were based on an upper limit of 15 mm for the nodal diameter of the normal long axis in cases of jugulodigastric and submandibular nodes and 10 mm for all other cervical nodes except for level VI (12).

The initial surgical procedure was either a bilateral procedure (near-total or total thyroidectomy) or a unilateral procedure (lobectomy) plus bilateral or ipsilateral central compartment dissection according to the ATA guidelines (9). Lateral lymph node dissection was performed if the patients satisfied at least one of the selection criteria, for example if there was a positive or suspicious radiographic finding in the lateral lymph nodes and multiple metastatic lateral lymph nodes were identified from the frozen biopsy. In this study, all patients underwent therapeutic lateral neck dissection that included levels II to V (13); level I was dissected only if there were radiography, cytopathology, or intraoperative findings suggestive of metastatic cancer, and no patient in this study underwent a level I dissection.

The clinical data were retrospectively analyzed with respect to gender, age, tumor size, tumor spread, presence of psammoma bodies, tumor multifocality, extrathyroidal extension (ETE), primary tumor location, central lymph node metastases, and lateral lymph node metastases. When multiple lesions were found in the specimen, the largest tumor or the most suspicious dominant nodule was analyzed.

### Statistical analysis

Statistics analysis was performed using Statistical Package for Social Sciences (SPSS, Inc., Chicago, IL, USA). Univariate analysis was performed using the chi-square test and Fisher’s exact test for categorical data. Non-parametric data with continuous variables were compared by the Mann–Whitney U test. Multivariate logistic regression analysis was performed to assess independent associations of LLNM with all factors found to be significant by univariate analysis, with adjustment for various established clinicopathological features. The results are presented as ORs with 95% confidence intervals and P values. Any P value less than 0.05 was considered statistically significant.

## RESULTS

In this study, 356 patients were enrolled. Among all patients, 278 patients were diagnosed with unilateral PTC and 78 patients with bilateral PTC. There were 89 males and 267 females with a male: female ratio of 1: 3. The age of the patients ranged from 12 to 85 years with a median age of 44.80 years. The tumor diameters ranged from 0.1 cm to 6.0 cm with a median diameter of 1.5 cm (Table 1). In this study, 344 patients underwent total thyroidectomy with bilateral or ipsilateral central compartment dissection plus ipsilateral lateral lymph node dissection, and 12 patients underwent total thyroidectomy with bilateral central compartment dissection plus bilateral lateral lymph node dissection.

**Table 1:** Patient demographics and tumor characteristics of 356 enrolled patients

Characteristics	Number of patients	Percentage (%)
Gender		
Female	267	75.00
Male	89	25.00
Age (y)		
≤ 25	30	8.43
25-35	74	20.79
35-45	102	28.65
45-55	86	24.16
55-65	43	12.08
> 65	21	5.90
Age (45y)		
≤ 45y	206	57.87
> 45y	150	42.13
Size (cm)		
≤ 0.5	37	10.39
0.5-1	83	23.31
1-1.5	69	19.38
1.5-2	54	15.17
> 2	113	31.74
Size (1.5 cm)		
≤ 1.5	189	53.09
> 1.5	167	46.91
Tumor spread		
Absent	283	79.49
Present	73	20.51
Psammoma bodies		
Absent	343	96.35
Present	13	3.65
Multifocality		
1	303	85.11
> 2	53	14.89
ETE		
Absent	244	68.54
Present	112	31.46
Unilateral or bilateral		
Unilateral	278	78.09
Bilateral	78	21.91
Primary location		
Superior	128	35.96
Middle	125	35.11
Inferior	58	16.29
Whole	45	12.64
Location superior		
Superior	128	35.96
Rest	228	64.04
CLNM		
Absent	109	30.62
Present	247	69.38
LLNM		
Absent	89	25.00
Present	267	75.00

ETE: extrathyroid extension; CLNM: central lymph node metastases; LLNM: lateral lymph node metastasis.

The clinicopathological features of the 356 patients enrolled in this study are shown in Table 1. Approximately, 75.0% (267/356) of patients demonstrated LLNM. Twelve patients underwent bilateral neck dissection in one operation, and 10 patients had bilateral lateral lymph node metastasis. There were 247 patients with CLNM (69.4%). In the LLNM positive group, 45 (16.9% within LLNM) patients were without CLNM and had skip metastasis.

In the univariate analysis, LLNM was significantly associated with age, tumor size, tumor spread, ETE, primary tumor location and central lymph node metastasis (p < 0.05), and no significant association was found between LLNM and gender, presence of psammoma bodies, multifocality, and unilateral or bilateral lesions (p > 0.05) (Table 2).

**Table 2:** Relationship between clinical and histological features of lateral lymph node metastasis (univariate analysis result)

Characteristics	LLNM negative	LLNM positive	Percentage (%)	Total	p
Gender					
Female	70	197	73.78	267	0.359
Male	19	70	78.65	89	
Age (y)					
≤ 25	2	28	93.33	30	0.002*
25-35	15	59	79.73	74	
35-45	25	77	75.49	102	
45-55	25	61	70.93	86	
55-65	13	30	69.77	43	
> 65	9	12	57.14	21	
Age (45y)					
≤ 45y	42	164	79.61	206	0.019*
> 45y	47	103	68.67	150	
Size (cm)					
≤ 0.5	20	17	45.95	37	0.000*
0.5-1	27	56	67.47	83	
1-1.5	16	53	76.81	69	
1.5-2	11	43	79.63	54	
> 2	15	98	86.73	113	
Size (1.5 cm)					
≤ 1.5	63	126	66.67	189	0.000*
> 1.5	26	141	84.43	167	
Tumor spread					
Absent	81	202	71.38	283	0.003*
Present	8	65	89.04	73	
Psammoma bodies					
Absent	87	256	74.64	343	0.422
Present	2	11	84.62	13	
Multifocality					
1	77	226	74.59	303	0.668
> 2	12	41	77.36	53	
ETE					
Absent	76	168	68.85	244	0.000*
Present	13	99	88.39	112	
Unilateral or bilateral					
Unilateral	71	207	74.46	278	0.657
Bilateral	18	60	76.92	78	
Primary location					
Superior	9	119	92.97	128	0.001*
Middle	42	83	66.40	125	
Inferior	33	25	43.10	58	
Whole	5	40	88.89	45	
Location superior					
Superior	9	119	92.97	128	0.000*
Rest	80	148	64.91	228	
CLNM					
Absent	64	45	41.28	109	0.000*
Present	25	222	89.88	247	

* Symbol for p < 0.05 in the univariate analysis. ETE: extrathyroid extension; CLNM: central lymph node metastases; LLNM: lateral lymph node metastasis.

In this study, the clinicopathological features of age, size and primary tumor location, were divided into categories as follows: Age ≤ 45 vs. > 45 years, tumor size ≤ 1.5 cm vs. > 1.5 cm, and tumor location superior versus elsewhere. With these categories, the patients were regrouped for further binary logistic regression analysis. The LLNM rate was higher for patients with age ≤ 45 y, larger tumor size, ETE, superior tumor location and positive CLNM. However, in the multivariate analysis, LLNM were significantly associated with primary tumor location, central lymph node metastasis (p < 0.05) and tumor size > 1.5 cm, with p = 0.05 (Table 3).

**Table 3:** Multivariate logistic regression analysis for the identification of predictive factors for lateral lymph node metastasis

Predictive factors	OR	95% CI OR		p

		Lower	Upper	
Size (≤ 1.5 cm versus > 1.5 cm)	2.062	1.000	4.249	0.050
Location (superior versus rest)	0.079	0.033	0.188	0.000
CLNM (absent versus present)	12.391	6.315	24.312	0.000

Only predictive factors from the multivariate logistic regression model. OR: odds ratio; CI: confidence interval.

## DISCUSSION

PTC is the major histopathology type of thyroid carcinoma and originates from thyroid follicular cells. Generally, it has a favorable long-term outcome, with a 10-year survival rate higher than 97% as an indolent disease (14). However, this type of cancer frequently metastasizes to lymph nodes (2-4). The pattern of lymphatic spread from the thyroid is relatively consistent, and the spread of lymph node metastasis in PTC takes place in a stepwise fashion. Cervical lymph node metastasis in PTC involves the central compartment first, followed by the ipsilateral lateral compartment and then the contralateral lateral compartment and the mediastinal lymph node (8), and recurrence is most likely to occur in the cervical lymph nodes (15). A study of the Surveillance, Epidemiology, and End Results database (SEER) found, among 9904 patients with PTC, that LNM was one of the significant predictive factors for poor outcome on multivariate analysis (9,16,17). Therefore, special attention should be paid to PTC patients with LNM, and the preoperative detection of LNM is important for reducing recurrence and improving prognosis (18,19).

The ATA guidelines advocate compartment-oriented en-bloc lateral neck dissection in patients with clinical LLNM, but offer no recommendation concerning which nodal levels should be dissected (9). A formal modified radical neck dissection including levels I, II, III, IV and V is usually not necessary in PTC patients with lateral compartment lymph node metastases because of the rarity of metastatic nodes at level I. Several authors have reported that PTC metastasis is generally present at levels II through V in lateral compartment neck metastasis, with level III or IV nodes consistently the most frequently involved, and comprehensive neck dissection including levels II to V is necessary for complete clearance of lateral neck metastasis (3). Level I dissection was excluded in the routine procedure; we performed level I dissection only with clinically apparent lymph nodes in level I, for example with a cytopathologically positive node, and because no patient had level I positive cytopathology, no patient underwent level I dissection. In this study, all the patients underwent a formal modified radical neck dissection including levels II (IIa and IIb), III, IV and V (Va and Vb) (13).

The cervical lymph node was involved in approximately 18%-90% of patients with PTC (2-4,6). The central compartment lymph node is the most common site of metastasis in PTC, and most patients with lateral compartment lymph node metastasis also have central compartment lymph node metastasis (20). However, 69.4% of patients in this study had CLNM, and 75% of patients had LLNM, which is consistent with results of previous studies, and 45 patients had skip metastasis (16.9% and 12.6% within the LLNM positive group and among all patients, respectively). In this study, all of the patients were suspiciously cN+, which included radiographic, cytopathologic, or intraoperative findings suggestive of metastases, so therapeutic lateral neck dissection was performed, which could explain why the metastasis rate of the lateral compartment was higher than that of the central compartment. In this study, 25% of patients did not have LLNM. All the patients underwent PE, US and contrast CT examination preoperatively, but fine needle aspiration examination was not systematically performed in our hospital at time of the study. When a palpable lateral lymph node is considered a metastatic lymph node, the false positive and false negative rate are both in the range of 20%-30% (21). Using US as a preoperative diagnostic tool for determining lateral lymph node status, the false positive rate becomes 1.9% and the false negative rate becomes 2.9% (22). However, US is overly operator-dependent and cannot be reviewed by one radiologist retrospectively. There are many US operators with different diagnostic experience and different US-clinical pathologic coincidence rates in our institute. In Kim’s report (23), the overall sensitivity, specificity, accuracy, positive predictive value, and negative predictive values for CT were 62%, 93%, 81%, 84%, and 80%, respectively. When US was combined with CT, these values were 66%, 88%, 79%,77%, and 81%, respectively. Therefore, the US/CT combination was superior to US alone in detection of metastatic lymph nodes in lateral neck levels, as suggested by other studies (23). In this research, if there were any suspicious radiographic findings (CT or US), the neck dissection could be conducted with or without a fine needle aspiration examination; 54 patients underwent FNA examination of lateral lymph nodes (15.2%), and 50 patients had a positive result and demonstrated LLNM in the final pathological examination (18.7%). Only 4 patients had an FNA examination with a uncertain result such as dysplasia or blood components in the non-LLNM group (4.5%), which could explain why 25% of patients did not have LLNM. A lateral neck dissection was unnecessary for those patients. Therefore, an exhaustive evaluation of the lateral lymph nodes might be necessary prior to surgery, and some aggressive treatment could be avoided.

The frequency of skip metastasis in PTC is approximately 2%-38% (24,25). In our study, the skip metastasis rate was similar to that of previous studies. Because lymph nodes in the lateral compartment of the neck can have metastasis even without central cervical lymph node involvement, the presence of lateral compartment lymph node metastasis should be fully examined by ultrasonography or radiography before surgery even if patients are found to have no central cervical lymph node metastasis during follow-up.

In this study, the positive central compartment lymph node metastasis group had a higher risk of lateral lymph node metastasis than did the negative group (p = 0.000, OR = 12.391, 95% CI 6.315-24.312, Table 3), which is in agreement with the results of most previous studies (26,27). The presence of CLNM warrants greater attention in LLN following thyroidectomy and CLN dissection. LLNM is associated with high rates of recurrence and distant metastasis and should be managed more aggressively when it is suspicious (28). Therefore, given the higher risk of LLN involvement, lateral compartment dissection might be considered a choice for these CLNM positive patients or for those with no less than 2 CLNM (29). In addition, if a lateral dissection is not performed for these patients, special inspection of the LLNs may be recommended during follow-up, and radioiodine may also be needed. Therefore, for those pathologically proven CLNM-negative PTC patients, a “wait and see” policy for LLN is probably adequate.

The upper part of the thyroid lobe (upper location) was an independent predictive factor for lateral LNM (p = 0.000, OR = 0.079, 95% CI 0.033-0.188, Table 3), which was similar to the results of other reports (27). This result may be explained by the hypothesis that the carcinoma cells from the upper region are more likely to be transported to the lateral lymph nodes by lymphatic flow along the superior thyroid artery (30,31). Therefore, if the primary tumor is located in the upper part of the thyroid lobe, more careful assessment should be performed before surgery.

Tumor size was an important prognostic feature of lateral lymph node metastasis (4,26). In this study, in the univariate analyses (Table 2), the rate of LNM increased obviously with the increase of the primary tumor size in a certain range, and the LNM rate was significantly higher in the group with Φ > 1.5 cm compared to those with Φ ≤ 1.5 cm (𝑃 < 0.000, Table 2), indicating that the primary tumor size is a significant factor associated with lateral compartment lymph node metastasis. However, the multivariate analyses did not show a strong association between the primary tumor size and lateral compartment lymph node metastasis, with p = 0.05. It may be difficult to use this numerical result in clinical practice. At same time, even if the size were small, more invasive cancers tend to have more lymph node metastasis. Since all of these tumor characteristics can be confirmed in the pathological diagnosis only after surgery, it may be quite difficult to know these data before the surgery procedure. Therefore, careful preoperative evaluation must be performed to confirm the presence of lateral cervical lymph node metastasis and to decide on the extent of the surgery.

It has been shown in several studies that males were more susceptible to PTC. Glattre and Kravdal considered that there was a significant correlation between the incidence of PTC and the estrogen level in women, whereas male patients were more vulnerable to unhealthy lifestyles and harmful environmental factors, such as smoking and drinking (32). In our study, in the univariate and multivariate analyses, there was no significant difference in the rate of LLNM between males and females (p > 0.05, Tables 2 and 3). Many staging or prognosis rating systems list age as one of the predictive factors of PTC prognosis (33). Our study shows a similar result; in the univariate analysis, the rate of LLNM metastasis in the age group ≤ 45 years was significantly higher than in the age group > 45 years (*P* < 0.01) (Table 2), and the age group ≤ 25 years had 93.33% LLNM, suggesting that PTC patients younger than 45 years may be more susceptible to LLNM. However, in the multivariate analysis, there was no association between age and LLNM.

In Ito’s report, massive extrathyroid extension was recognized as an independent prognostic factor for relapse-free survival (P = 0.0003), and minimal extension was not (34,35). Massive extrathyroidal extension has been reported as an important risk factor for CLNM and LLNM, and it has a negative effect on survival rate. In this study, the LLNM rate significantly increased with extrathyroidal extension in the univariate analysis (p = 0.000, Table 2). However, in multivariate analysis, extrathyroidal extension as a risk factor did not demonstrate a significant association with the LLNM rate (p = 0.094, Table 3), perhaps because the extrathyroid extension was not massive enough.

Multifocality and bilaterality of the tumor was not significantly associated with lateral lymph node metastasis in the univariate and multivariate analysis in this study. In Lin’s report, it is important to distinguish between two types of multifocal tumors: multifocal-independent primary tumors and tumors with an intrathyroid metastases-like tumor spread in the thyroid gland; the latter was frequently associated with lateral lymph node metastases (36). In the univariate analysis, tumor spread was significantly associated with LLNM, with p = 0.003 (Table 2), but not in the multivariate analysis (p = 0.105, Table 3). This difference was possibly caused by the study limitations of an inadequately large sample size and a single-center research design.

There were several potential limitations to this study. Lee suggested that the number of positive central lymph nodes is significantly associated with tumor size, and patients with ≥ 3 positive central lymph nodes are more likely to have extrathyroidal extension (28). Our study only investigated lateral lymph node metastasis; we did not provide data on other clinicopathological features or long-term follow-up results, such as disease recurrence, postoperative radioiodine studies, thyroglobulin levels, thyroid stimulating hormone levels, and disease-free survival. We are currently collecting full clinicopathological data and long-term follow-up results for a consecutive report that could be used to improve clinical practice.

In conclusion, patients with PTC, with the primary tumor located in the upper part of the thyroid lobe, with positive central compartment lymph node metastasis and with tumor size > 1.5 cm in diameter, are more likely to have lateral lymph node metastasis, and careful preoperative evaluation should be performed before surgery. The other clinicopathological features, such as age, tumor spread, and ETE, demonstrated significant differences in the univariate analysis, but not in the multivariate analysis, perhaps due to the small sample size and single-center limitations. Therefore, larger multicenter and long-term follow-up studies are needed to better answer questions regarding the impact of these metastases on prognosis and survival.
